# Long-term residential sunlight exposure associated with cognitive function among adults residing in Finland

**DOI:** 10.1038/s41598-022-25336-6

**Published:** 2022-12-02

**Authors:** Kaisla Komulainen, Christian Hakulinen, Jari Lipsanen, Timo Partonen, Laura Pulkki-Råback, Mika Kähönen, Marianna Virtanen, Reija Ruuhela, Olli Raitakari, Suvi Rovio, Marko Elovainio

**Affiliations:** 1 Department of Psychology and Logopedics, University of Helsinki, P.O. Box 21, 00014 Helsinki, Finland; 2 Finnish Institute for Health and Welfare, Helsinki, Finland; 3 Department of Clinical Physiology, Tampere University Hospital and Faculty of Medicine and Health Technology, Tampere University, Tampere, Finland; 4 School of Educational Sciences and Psychology, University of Eastern Finland, Joensuu, Finland; 5 Division of Insurance Medicine, Karolinska Institutet, Stockholm, Sweden; 6 Weather and Climate Change Impact Research, Finnish Meteorological Institute, Helsinki, Finland; 7 Centre for Population Health Research, University of Turku and Turku University Hospital, Turku, Finland; 8 Research Centre of Applied and Preventive Cardiovascular Medicine, University of Turku, Turku, Finland; 9 Department of Clinical Physiology and Nuclear Medicine, Turku University Hospital, Turku, Finland; 10 Research Program Unit, Faculty of Medicine, University of Helsinki, Helsinki, Finland

**Keywords:** Human behaviour, Climate sciences, Cognitive ageing, Learning and memory

## Abstract

While sunlight may influence cognitive function through several pathways, associations of residential sunlight exposure with cognitive function are not well known. We evaluated associations of long-term residential sunlight exposure with cognitive function among a representative cohort of 1838 Finnish adults residing in Finland who underwent comprehensive cognitive assessment in midlife. We linked daily recordings of global solar radiation to study participants using residential information and calculated the average daily residential exposure to sunlight for four exposure time intervals: 2 months, 1 year, 2 years and 5 years prior to the date of the cognition assessment. Associations of the residential sunlight exposure with cognitive function were assessed using linear regression analyses adjusting for season, sex, age, and individual- and neighborhood-level socioeconomic characteristics. Greater average residential sunlight exposure over 2 and 5 years prior to the cognitive function assessment was associated with better global cognitive function (b = 0.13, 95% CI = 0.01, 0.25; b = 0.17, 95% CI = 0.05, 0.29, per 1 MJ/m^2^ difference in sunlight exposure), while no associations with global cognitive function were observed at shorter exposure time intervals. In domain-specific analyses, greater residential exposure to sunlight over 1, 2 and 5 years prior to the cognitive function assessment was associated with better performance on new learning and visual memory (b = 0.10, 95% CI = 0.00, 0.20; b = 0.16, 95% CI = 0.04, 0.28; b = 0.19, 95% CI = 0.08, 0.31) and sustained attention (b = 0.15, 95% CI = 0.05, 0.25; b = 0.18, 95% CI = 0.06, 0.30; b = 0.17, 95% CI = 0.05, 0.29), but worse performance on reaction time (b =  − 0.12, 95% CI =  − 0.22, − 0.02; b = -0.15, 95% CI =  − 0.28, − 0.02; b =  − 0.18, 95% CI =  − 0.30, − 0.05). Residential sunlight exposure was not associated with executive function. These findings suggest long-term residential sunlight exposure may be an environmental factor influencing cognitive function among a cognitively healthy cohort residing in Northern Europe. Further studies in populations residing in different geographical locations are needed.

## Introduction

Sunlight is implicated in cognitive function through several pathways. Sunlight exposure is a key determinant of the circadian rhythms and sleep–wake schedule^1,2^, and altered sleep is known to affect several domains of cognitive function^3–5^. Disruption in circadian rhythms as well as the lack of sunlight have been associated with fluctuations in mood and emotion regulation^6–8^, which in turn may have both short-term^9–11^ and longer-term effects on cognitive performance^12^. Experimental evidence has suggested physiological pathways through which sunlight may influence cognitive function. In mice, short-term moderate exposure to ultraviolet (UV) radiation which includes non-visible wavelengths of sunlight produces reversible beneficial effects on learning and memory through triggering glutamate release at presynaptic axon terminals in the motor cortex and hippocampus^13^. Exposure to UV radiation has also been hypothesized to affect brain by altering proteins, peptides and small molecules, such as β-endorphin, vitamin D and nitric oxide, in the skin or circulating blood^14–17^.

While experimental findings among humans suggest that short-term solar radiation exposure over a few hours, especially when directed to the head and neck, has adverse effects on cognitive function^18,19^, observational data on longer-term residential exposure to natural sunlight suggest a protective association of more sunlight against the risk of cognitive decline. A cohort study among over 16,000 participants in the U.S. found a dose–response association of lower 2-week average solar radiation exposure with greater odds of cognitive impairment, but only among a subpopulation of participants who were depressed^20^. Another study in the same U.S. cohort observed an association of lower 1-year average solar radiation exposure with incident cognitive decline^21^. In a Chinese elderly population, participants reporting greater habitual sun exposure were more likely to perform better in the Mini-Mental State Examination measuring global cognitive function^22^.

Previous observational studies assessing the associations of sunlight with cognitive function have been conducted in predominantly elderly populations and with crude measures of cognitive decline^20–22^. Cognitive decline is yet a gradual process, and risk factors of decline can occur long before any clinically significant impairment is detected. Better cognitive function in young adulthood and midlife has been associated with less steep cognitive decline in older age^23–27^, and identifying determinants for young adult and midlife cognitive function is thus important in terms of primordial prevention of cognitive decline. It is not yet known whether long-term residential sunlight exposure is associated with cognitive function in young adulthood and midlife before the clinical signs of decline manifest. Using prospective data among adults residing in Finland, we assessed the associations of long-term residential sunlight exposure with cognitive function among participants who underwent a comprehensive cognitive assessment in midlife. Finland is a Northern European country extending 1200 km from north to south (60–70° N), where the levels of sunlight differ considerably across regions.

## Methods

### Study sample

Data were from the Cardiovascular Risk in Young Finns Study (YFS) which is a nationwide prospective cohort study on cardiovascular risk factors from childhood to adulthood^28^. The initial sample included 3596 participants aged 3–18 years in 1980. For this study, we included 1935 participants who had data on the date of the outcome (cognitive function) assessment in the 2010–2012 data collection phase, and data on their residence on each day 5 years prior to the outcome assessment date. Of these 1935 participants, 13 were excluded owing to missing data on individual-level or neighborhood-level socioeconomic factors, and an additional 84 were excluded owing to missing data on all cognitive tests, resulting in 1838 participants. All available data for each cognitive test were used in the analyses. Due to technical reasons or refusal to participate in some of the cognitive tests, the sample sizes between analyses on different cognitive domains varied between 1635 and 1838 participants. The study was conducted according to the Declaration of Helsinki and was approved by the Ethics Committee of the Hospital District of Southwest Finland. All participants gave written informed consent.

### Cognitive function

In 2011, participants aged 34 to 49 underwent the Cambridge Neuropsychological Test Automated Battery (CANTAB, Cambridge Cognition, Cambridge, UK). The YFS test battery included four tests assessing different domains of cognitive function: visual memory and associative new learning (Paired Associates Learning test), reaction and movement time (Reaction Time test), visual processing and sustained attention (Rapid Visual Information Processing test) and short-term working memory and executive control (Spatial Working Memory test). Each of these four tests comprised several variables. As previously described^29^, principal component analysis was conducted in the complete cognitive function data, and the first component resulting from this analysis was considered an indicator of global cognitive function. Domain-specific performance in each four cognitive domains was assessed as the first principal component resulting from test-specific principal component analyses^29,30^. The principal components were rank-order normalized, which resulted in four normally distributed components (mean = 0, SD = 1), and transformed so that greater values in each component indicated better cognitive function (including the Reaction Time test, where greater values thus indicated shorter reaction times). The detailed CANTAB protocol and validation of the cognitive data in the YFS are presented elsewhere^29,30^.

### Sunlight exposure

Meteorological data on sunlight were obtained from the Finnish Meteorological Institute, where spatial mean values of 10 km × 10 km gridded daily data on global solar radiation (MJ/m^2^) were calculated for each residential zip code area^31^. We obtained detailed residential history for each participant from Statistics Finland, and linked the solar radiation data to the participants’ zip code on each day prior to the cognition assessment. Then, we calculated the average daily exposure to global solar radiation prior to the date of the cognition assessment for four different exposure lengths: 2 months, 1 year, 2 years and 5 years.

Seasons (winter December to February, spring March to May, summer June to August and fall September to November) were determined based on the date of the outcome assessment. Educational attainment (highest level of educational attendance or completed education) was measured through self-reports in 2010–2012, categorized into four ascending groups: primary and lower secondary education, upper secondary education, Bachelor’s degree program or equivalent; Master’s degree program or higher. Three neighborhood-level socioeconomic characteristics in each zip code were obtained from the records of Statistics Finland (Paavo postal code area statistics): proportion of residents with a college degree, proportion of unemployed residents and mean income per resident. These were linked to the participants based on their residential history.

### Statistical analysis

The associations of the residential sunlight exposure with cognitive function were assessed with linear regression analyses using ordinary least squares estimation. We conducted separate analyses for global cognitive function and the four specific cognitive domains (visual memory and associative learning, reaction time, visual processing and sustained attention, short-term working memory and executive control) at each four exposure lengths (2 months, 1 year, 2 years, 5 years). We first estimated the associations of residential sunlight exposure with cognitive function adjusting for the season of the outcome assessment, sex and age. These models were then further adjusted for individual-level educational attainment, the proportion of residents with a college degree in the neighborhood, the proportion of unemployed residents in the neighborhood, and the mean income per resident in the neighborhood. A *p*-value of > 0.05 was considered significant in a 2-sided test. A power calculation indicated that with our smallest sample size of 1635, we had 90% power to detect an effect size of 0.013. All analyses were conducted using Stata version 17.0 and R version 4.2.1.

### Ethics approval and consent to participate

The study was conducted according to the Declaration of Helsinki and was approved by the Ethics Committee of the Hospital District of Southwest Finland. All participants gave written informed consent.

## Results

Characteristics of the 1838 participants are presented in Table 1. There were 1025 (56%) women, and the mean age was 41.9 years (SD = 5.0) at the time of the cognitive function assessment. Average daily residential exposure to global solar radiation over one year prior to the cognitive function assessment was 9.4 MJ/m^2^ (SD = 0.5). The distributions of residential sunlight exposures of different lengths are presented in Supplementary Fig. 1. Correlations between global cognitive function and the four specific cognitive domains are shown in Supplementary Table 1. Supplementary Fig. 2 presents the geographical variation in average daily global solar radiation in Finland in 2011.

**Table 1:** Characteristics of 1,838 participants from the Cardiovascular Risk in Young Finns study.

	Mean (SD)	n [%]	N
Sex (female)		1025 [56%]	1838
**Age**	41.9 (5.0)		1838
**Season**			1838
Winter		575 [31%]	
Spring		615 [33%]	
Summer		227 [12%]	
Fall		421 [23%]	
**Individual-level educational attainment in 2011**			1838
Primary or lower secondary		56 [3%]	
Upper secondary		1070 [58%]	
BA		392 [21%]	
MA or higher		320 [17%]	
Educational attainment in neighborhood^a,b^	10.4 (3.5)		1838
Unemployment in neighborhood^a,b^	4.9 (1.8)		1838
Mean income in neighborhood (EUR)^a^	23,451.7 (4171.2)		1838
**Global solar radiation (MJ/m**^**2**^**)**			1838
2-month daily average	7.5 (6.2)		
1-year daily average	9.4 (0.5)		
2-year daily average	9.3 (0.4)		
5-year daily average	9.1 (0.5)		
**Global cognitive function in 2011**	0.0 (1.0)		1635
Paired Associates Learning	0.0 (1.0)		1670
Reaction Time	0.0 (1.0)		1656
Rapid Visual Information Processing	0.0 (1.0)		1802
Spatial Working Memory	0.0 (1.0)		1838

^a^Values are 1-year daily averages based on participants' residential information.

^b^Educational attainment and unemployment in neighborhood were calculated based on the proportion of participants with a college degree and proportion of participants unemployed in neighborhood (zip code area).

**Figure 1:**
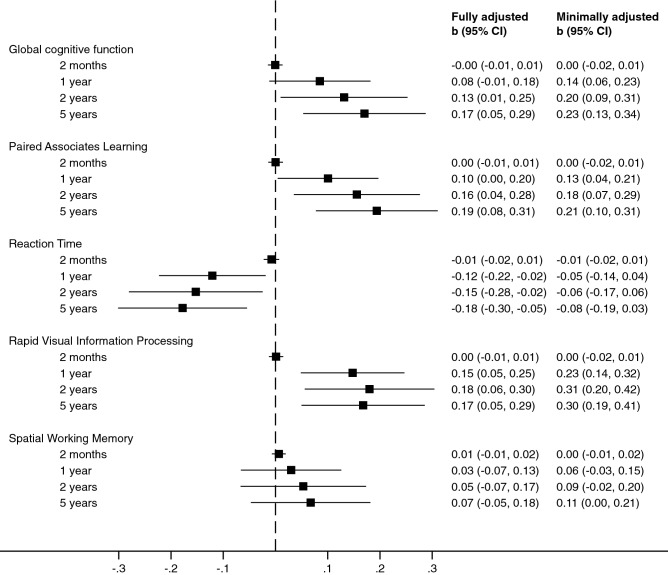
Associations of long-term sunlight exposure with cognitive function in the Cardiovascular Risk in Young Finns Study Abbreviations: CI, confidence interval. Estimates are differences in the rank-order normalized cognitive function scores per 1 MJ/m^2^ difference in the average daily exposure to global solar radiation in residential neighborhood from linear regression analysis.

Figure 1 shows the associations of the average daily residential sunlight exposure with global cognitive function and the four cognitive domains. The average 2-month residential exposure to sunlight was not associated with any of the cognitive function outcomes when adjusting for season, sex and age. These results remained unchanged after further adjustment for individual-level educational attainment, the proportion of residents with a college degree in the neighborhood, the proportion of unemployed residents in the neighborhood, and the mean income per resident in the neighborhood.

Instead, greater average residential exposure to sunlight at exposure lengths of 1 year or longer was associated with better performance on global cognitive function, visual memory and associative learning (Paired Associates Learning) as well as visual processing and sustained attention (Rapid Visual Information Processing) in models adjusted for season, sex and age. These associations were somewhat attenuated but remained statistically significant after adjustment for individual- and neighborhood-level socioeconomic factors, except that the association of 1-year average residential sunlight exposure with global cognitive function was no longer significant (*p* = 0.09). No associations between the average residential sunlight exposure and executive control (Spatial Working Memory) were observed either in the minimally or the fully adjusted models. The average residential sunlight exposure at exposure lengths of 1 year or longer was not associated with reaction time (Reaction Time) in the models adjusted for season, sex and age. However, we observed an inverse association between greater average residential sunlight exposure at exposure lengths of 1 year or longer and reaction time after additional adjustment for individual-level and neighborhood-level socioeconomic factors (Fig. 1).

## Discussion

In this population-based study among 1,838 adults residing in Finland, we observed associations of long-term residential sunlight exposure with cognitive function. Overall, greater long-term exposure to sunlight in residential neighborhood was associated with better global cognitive function, but the associations of sunlight with cognitive function were also domain-specific; greater residential sunlight exposure was associated with better performance in cognitive tasks requiring visual memory, new learning and sustained attention, while no association with spatial working memory and an inverse association with reaction time performance was observed. The associations were small in magnitude, but robust to adjustment for individual-level as well as for neighborhood-level confounders. In our data, the differences between the highest and lowest quintile averages in the residential sunlight exposure at exposure lengths of 1 year or longer were from 1.2 to 1.4 MJ/m^2^. There is a linear decline in global cognitive function of 0.05 SD per year between ages 34–49 in the YFS^29^, and thus the observed differences in cognitive function between participants in the highest and lowest 20% of residential sunlight exposure correspond roughly to a 2- to 4-year difference in cognitive age.

While direct short-term solar radiation exposure over a few hours has been shown to impair cognitive functioning in humans^18,19^, longer-term residential or habitual exposure to natural sunlight has been associated with a lower risk of cognitive decline in elderly populations^21,22^. Similarly, we observed that residence in areas receiving more sunlight was associated with better global cognitive function also among young and middle-aged adults (aged 34–49 years). These associations were observed when we assessed the average residential exposure to sunlight over 2 years and 5 years prior to the cognitive function measurement, and they were robust to adjustment for season, sex, age as well as individual- and neighborhood-level socioeconomic factors. At shorter exposure time intervals, i.e. 2 months and 1 year prior to the cognitive function assessment, we found no associations of residential sunlight exposure with global cognitive function, suggesting that the association of residential sunlight exposure with global cognition is small in magnitude but it may accumulate over time.

In domain-specific analyses, average residential sunlight exposure over 2 months prior to the cognitive assessment was not associated with cognitive function. Instead, greater residential exposure to sunlight over 1 year, 2 years and 5 years prior to the cognitive assessment was associated with better performance in terms of visual memory and new learning as well as visual processing and sustained attention. In contrast, greater residential exposure to sunlight was unexpectedly associated with worse performance in terms of reaction time in models adjusted for individual- and neighborhood-level socioeconomic factors in addition to season, sex and age. Prior analyses in the YFS also observed some evidence of associations of cardiovascular risk factors and reaction time in an unanticipated direction (i.e. risk factors associated with better reaction time performance)^32,33^, and correlations of reaction time with other domains of cognitive function were relatively low in our data. These observations may be partially due to reaction time being a reactive function which is dependent on peripheral innervation in addition to the operation of the central nervous system, and thus reaction time may not represent cognitive function similarly to the other cognitive domains we assessed. Nevertheless, the unexpected direction of association between residential sunlight exposure and reaction time we observed needs to be interpreted with caution.

In contrast to associations with other cognitive domains, residential sunlight exposure was not associated with performance in the Spatial Working Memory test, which is considered a sensitive measure of functions related to the frontal lobe, such as executive function. Given that executive function plays a role also in new learning, sustained attention, and visual memory, it is not clear whether our findings reflect true domain-specificity. However, if long-term residential sunlight exposure was particularly important to new learning, sustained attention and visual memory, and less so to executive function, such domain-specificity may help elucidate mechanisms explaining the associations of sunlight with cognition. While potential biological links in humans are still unknown, experimental evidence from mice showed that short-term UV radiation exposure had beneficial effects on learning and memory through elevated blood urocanic acid and increased synaptic release of glutamate into the motor cortex and hippocampus^13^. Although these short-term effects appear to be reversible^13^, it is possible that similar mechanisms are partially responsible for the long-term associations of residential sunlight exposure with new learning and memory also in humans^34^. Apart from other hypothetical biological pathways through which sunlight may affect the brain, such as the potential neuroprotective effects of greater levels of vitamin D^15,16,35^, the opioid receptor-mediated effects of elevated circulating β-endorphin^14^ or vasodilation due to nitric oxide alterations following exposure to UV radiation^17^, the associations of residential sunlight exposure with cognitive function may involve psychological mechanisms related to sleep^1–5,36,37^ or mood^6–12,38,39^, for instance. Determining whether such factors are particularly important to enhance performance on specific cognitive domains needs to be elucidated in further studies. More studies are also needed to evaluate whether long-term residential sunlight exposure is differently associated with specific domains of cognitive function in populations residing in different geographical locations, given that sunlight exposure is greatly dependent on latitude.

Main strengths of this study include the comprehensive assessment of cognitive function among relatively young participants from a representative population-based sample, and the use of detailed residential address information to link the daily meteorological data to each participant, which allowed us to calculate the sunlight exposures accurately for different exposure lengths. Some limitations should also be noted. Our data were from an observational cohort study, and our findings do not have a causal interpretation. Due to the complex relationships of individual- and area-level factors as well as regional differences in residential sunlight exposure, evaluating the associations of residential sunlight exposure with cognitive function is not straightforward. Although we controlled for a robust set of individual- and neighborhood-level confounders in the analyses, we cannot rule out potential bias due to residual or unmeasured confounding by geographically distributed factors that could affect cognitive function. In addition, residential exposure to sunlight does yet only approximate the actual exposure to sunlight, given that we did not have data on how much the participants spent time in their actual residential location or outdoors. The differences in cognitive function between participants in the highest and lowest 20% of residential sunlight exposure corresponded roughly to a 2- to 4-year difference in cognitive age in midlife. Although it is unclear to what extent such differences are clinically meaningful, comparable effect sizes have been reported with other exposures, such as daily smoking in adolescence or early adulthood^32^. Finally, our findings are based on a homogeneous population residing in a high-income country in Northern Europe. Given that sunlight exposure is greatly dependent on latitude, the generalizability of our findings to other populations, especially to those residing in different latitudes, is limited.

## Conclusions

We observed that a greater long-term exposure to sunlight in residential neighborhood was associated with better global cognitive function in midlife among adults residing in Finland. The associations of sunlight exposure with cognitive function were domain-specific, suggesting that long-term residential sunlight exposure is relevant to new learning, memory and sustained attention in particular. These findings suggest that residential sunlight exposure may be one environmental factor explaining interindividual variability in cognitive function among Northern European adults. Further studies should examine potential pathways explaining these associations, evaluate these findings in other populations residing in different latitudes, and assess the relevance of sunlight as well as other environmental exposures to cognitive health across different life stages.

## Supplementary Information

Supplementary Information.

## Data Availability

The dataset supporting the conclusions of this article were obtained from the Cardiovascular Risk in Young Finns study which comprises health related participant data. The use of data is restricted under the regulations on professional secrecy (Act on the Openness of Government Activities, 612/1999) and on sensitive personal data (Personal Data Act, 523/1999, implementing the EU data protection directive 95/46/EC). Due to these restrictions, the data cannot be stored in public repositories or otherwise made publicly available. Data access may be permitted on a case-by-case basis upon request only. Data sharing outside the group is done in collaboration with YFS group and requires a data-sharing agreement. Investigators can submit an expression of interest to the chairman of the publication committee, Prof Mika Kähönen (Tampere University, Finland) and Prof Terho Lehtimäki (Tampere University, Finland).
