# Supplementary material for: Long-term residential sunlight exposure associated with cognitive function among adults residing in Finland
Source: Sci Rep. 2022 Dec 2;12:20818. doi: 10.1038/s41598-022-25336-6 (PMC9718732; doi:10.1038/s41598-022-25336-6)
Supplement: Supplementary file 1 — Supplementary Information. [file 41598_2022_25336_MOESM1_ESM.pdf]

Long-term residential sunlight exposure associated with cognitive function among adults residing in Finland

Kaisla Komulainen, Christian Hakulinen, Jari Lipsanen, Timo Partonen, Laura Pulkki-Råback, Mika Kähönen, Marianna Virtanen, Reija Ruuhela, Olli Raitakari, Suvi Rovio and Marko Elovainio

Supplementary Information

Supplementary Figure S1. Distributions and quintile averages of the residential sunlight exposure at the different exposure lengths

Supplementary Figure S2. Geographical variation in average daily global solar radiation across zip code areas in Finland in 2011

Supplementary Table S1. Correlations between global cognitive function and specific domains of cognitive function among 1,635 participants from the Cardiovascular Risk in Young Finns Study

Supplementary Figure S1. Distributions and quintile averages of the residential sunlight exposure at the different exposure lengths

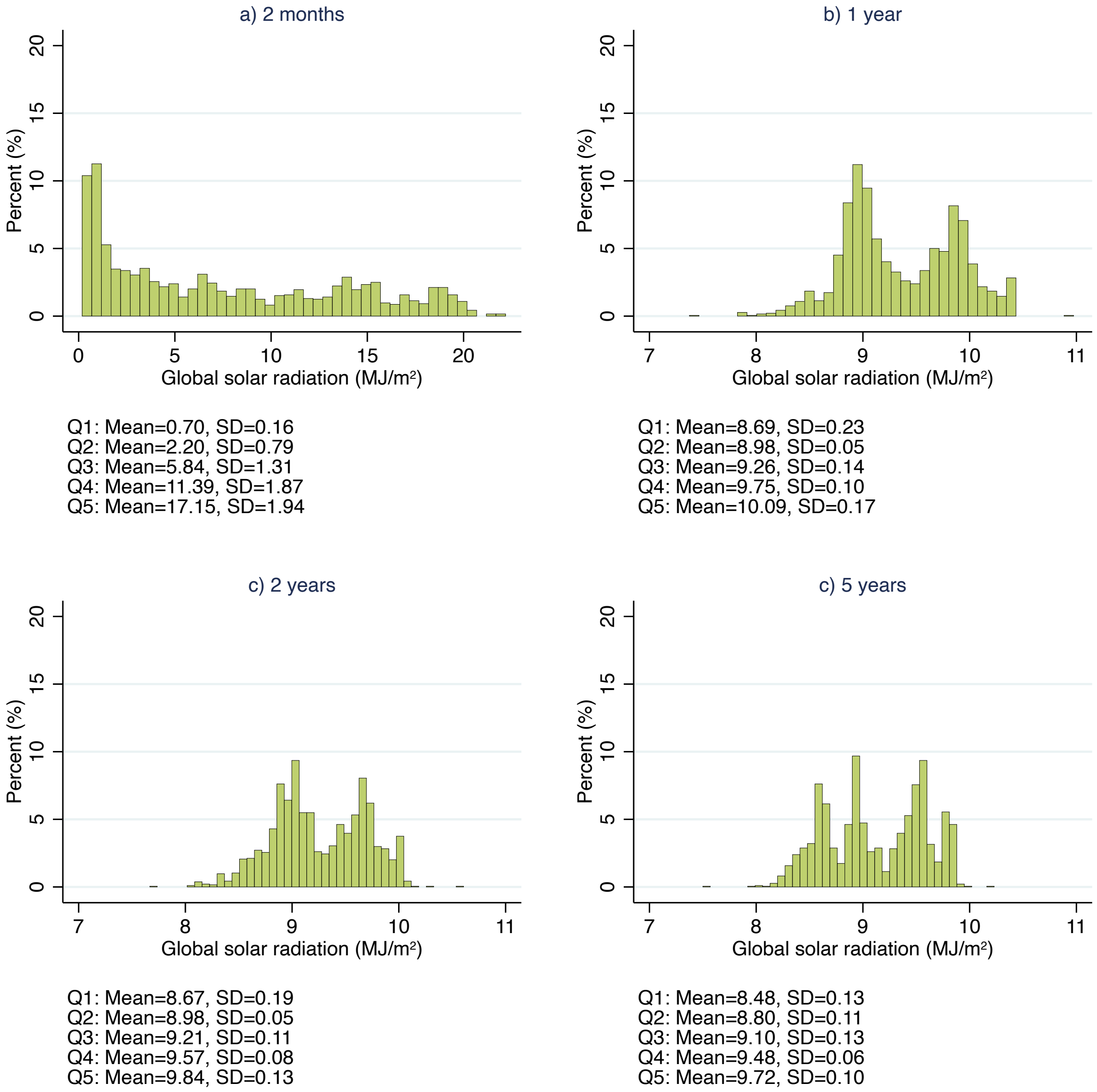

Abbreviations: Q1–Q5, 1st–5th quintile; SD, standard deviation

Supplementary Figure S2. Geographical variation in average daily global solar radiation across zip code areas in Finland in 2011

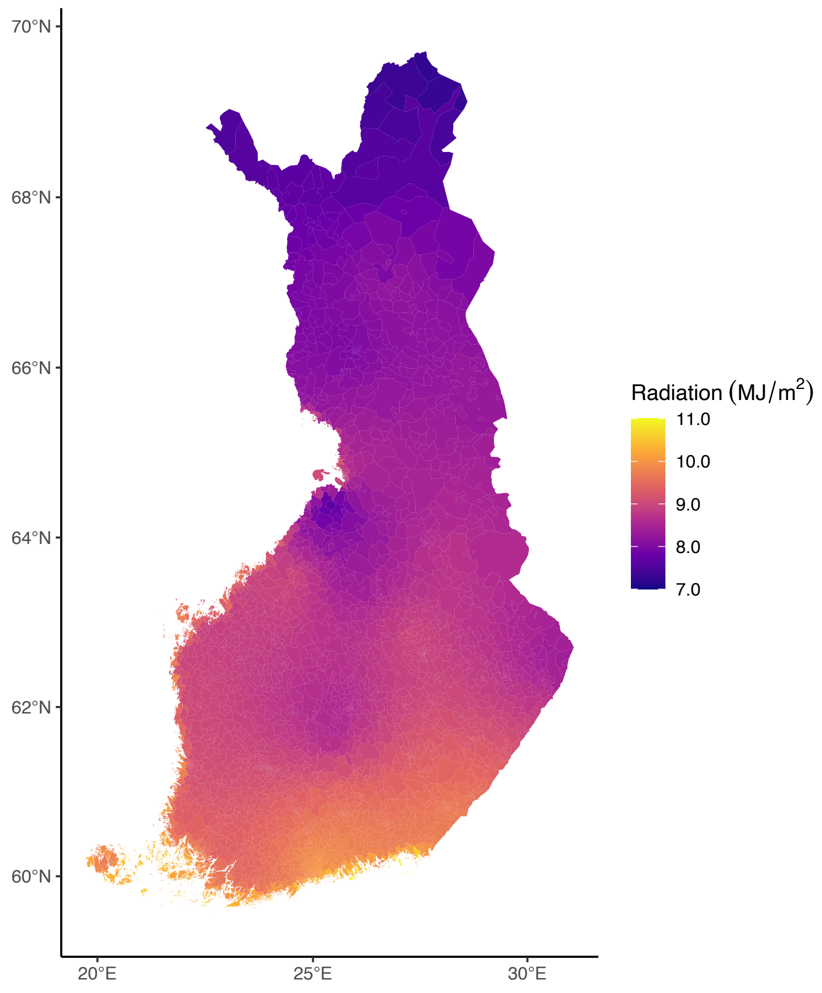

Supplementary Table S1. Correlations between global cognitive function and specific domains of cognitive function among 1,635 participants from the Cardiovascular Risk in Young Finns Study

	Global	PAL	RTI	RVP
Global				
PAL	0.7575			
RTI	0.1215	0.0588		
RVP	0.6161	0.2949	0.1043	
SWM	0.7433	0.2614	0.0780	0.3085

Values are Pearson's correlation coefficients (*r*). P-values of all correlations <0.05.

Abbreviations: Global, global cognitive function; PAL, Paired Associates Learning; RTI, Reaction Time; RVP, Rapid Visual Information Processing; SWM, Spatial Working Memory
